# Protein**-**Based Three-Dimensional Whispering-Gallery-Mode Micro-Lasers with Stimulus-Responsiveness

**DOI:** 10.1038/srep12852

**Published:** 2015-08-04

**Authors:** Yun-Lu Sun, Zhi-Shan Hou, Si-Ming Sun, Bo-Yuan Zheng, Jin-Feng Ku, Wen-Fei Dong, Qi-Dai Chen, Hong-Bo Sun

**Affiliations:** 1 State Key Laboratory on Integrated Optoelectronics, College of Electronic Science and Engineering, Jilin University, 2699 Qianjin Street, Changchun 130012,China; 2 College of Physics, Jilin University, 119 Jiefang Road, Changchun, 130023, China

## Abstract

For the first time, proteins, a promising biocompatible and functionality-designable biomacromolecule material, acted as the host material to construct three-dimensional (3D) whispering-gallery-mode (WGM) microlasers by multiphoton femtosecond laser direct writing (FsLDW). Protein/Rhodamine B (RhB) composite biopolymer was used as optical gain medium innovatively. By adopting high-viscosity aqueous protein ink and optimized scanning mode, protein-based WGM microlasers were customized with exquisite true 3D geometry and smooth morphology. Comparable to previously reported artificial polymers, protein-based WGM microlasers here were endowed with valuable performances including steady operation in air and even in aqueous environments, and a higher quality value (***Q***) of several thousands (without annealing). Due to the “smart” feature of protein hydrogel, lasing spectrum was responsively adjusted by step of ~0.4 nm blueshift per 0.83-mmol/L Na_2_SO_4_ concentration change (0 ~ 5-mmol/L in total leading to ~2.59-nm blueshift). Importantly, other performances including ***Q***, FWHM, FSR, peak intensities, exhibited good stability during adjustments. So, these protein-based 3D WGM microlasers might have potential in applications like optical biosensing and tunable “smart” biolasers, useful in novel photonic biosystems and bioengineering.

It has been drawing great efforts recently to utilize various natural-product-based/-derived biomacromolecules as biopolymers, for example, kinds of proteins and deoxyribonucleic acid (DNA), as important constituents or even core functional materials in multifarious photonic devices and systems^1^,^2^,^3^,^4^,^5^,^6^,^7^,^8^,^9^,^10^,^11^. For instance, chicken albumen dielectrics could be implemented in organic field-effect transistors (OFETs)^1^; three-dimensional (3D) photonic crystals were successfully constructed with silk fibroin^2^. Also in our previous work^3^,^4^,^5^, multifunctional microlenses were FsLDW-fabricated with bovine serum albumin (BSA). Moreover, DNA is widely used as a novel material for photonics^6^, especially, in light-emitting diodes (LEDs) to boost light emission^7^. Compared with synthetic polymer and hydrogel biomaterials relied more at present^12^,^13^, natural-product-based/-derived biomacromolecules are endowed with more comprehensive, particular, and even non-substitutable bio-related virtues. On one hand, photonic applications mentioned above were motivated by outstanding features of natural-product biomacromolecules such as biocompatibility, eco-friendliness, extensive and replenishable resources^1^,^2^,^3^,^4^,^5^,^6^,^7^. More significantly, on the other hand, a variety of special molecule structures and therefore biological, chemical, and physical properties of diversified biomacromolecules, which are irreplaceably resulted from the long-term natural evolution, enable functionalities like high optical transparency^2^,^6^,^7^,^8^,^9^, light emission enhancement^8^,^9^, “smart” environmental responsiveness^3^,^4^, bio-illumination^8^,^10^, tailorable soft mechanical characteristics^2^,^5^,^6^,^8^, biocatalysis^14^, and specific recognition^11^,^15^. Besides, facile and flexible functionalization is another significant advantage of biomacromolecule-based/-derived biomaterials via multi-methods like blending^11^, chemical modification^16^,^17^, and even gene-engineering customization^16^,^17^. Thus, it offers a huge “module library” of natural functional biomaterials to select from as needed for applications in various fields, for example, bionics of not only structures but also material-composition.

In particular, active photonics based on natural-product biopolymers have also been proto-attempted for above reasons, though it remains quite unexplored^8^,^9^,^10^. Silk-based two-dimensional (2D) photonic crystal films were doped with diverse active components, including dyes, quantum dots, and green fluorescent protein (GFP), for enhancing fluorescent emission^8^,^9^. Furthermore, a millimeter-scale Fabry-Perot (F-P) cavity based “biolaser” was demonstrated by placing GFP aqueous solution between a pair of dielectric mirrors^10^. Nevertheless, probably limited by the nature and therefore uneasy 3D-nanoprocessing of biomacromolecule materials, it has not been reported until now as far as we know to finely customize 3D whispering-gallery-mode (WGM) microlasers directly using natural-product biomacromolecules as host material. The existing WGM optical microcavities and microlasers are built generally with silicon dioxide (SiO_2_)^18^,^19^,^20^,^21^,^22^,^23^, semiconductors^24^,^25^, artificial polymers^26^,^27^, and synthetic organic crystals^28^. These WGM-based optical microdevices are of great advantages including facile integration, high ***Q*** (related to photo-confinement in the cavities), and high detection sensitivity^29^.

Herein, for the first time, we used proteins, a promising class of biocompatible and multifunctional natural biomacromolecules, as the host material to fabricate 3D WGM microlasers by multiphoton femtosecond laser direct writing (FsLDW, see Fig. 1). During laser processing, protein molecules (BSA) were photocrosslinked with the help of “self-photosensitization” of their own^30^,^31^ and probable photosensitization of rhodamine B (RhB)^32^,^33^,^34^,^35^. Then, the BSA/RhB composite biopolymer worked as optical active medium in as-formed 3D WGM microdisks. It was important for avoiding RhB dimerization and increasing fluorescent intensity that RhB molecules would site-selectively and discretely bind onto BSA biomacromolecules in aqueous solutions. That is, BSA/RhB composite biopolymer could be innovatively utilized as satisfactory photo-machinable optical gain medium. FsLDW, improved with high-viscosity aqueous protein ink and optimized scanning mode, ensured exquisite true 3D geometry and high-quality morphology (average roughness as low as ~5 nm) of protein-based 3D WGM microlasers. Consequently, steady and good operation under proper conditions in air and even in aqueous environments was experimentally proved. ***Q*** >2000 and full width at half maximum (FWHM) of ~0.25 nm were achieved in air even without annealing processing, better than similar WGM microlasers based on artificial polymers^27^. Even for protein-based 3D WGM microlasers operated in aqueous solutions, which was rarely reported previously using artificial polymers to our knowledge, ***Q*** was up to ~3300 and FWHM was as small as ~0.18 nm. Resulted from environment-stimulus response of protein hydrogels, the fine lasing spectrum could be adjusted for a ~2.59-nm blueshift gradually and responsively to Na_2_SO_4_ concentration variation from 0 to 5 mmol/L (about 0.4 nm *vs* 0.83 mmol/L per step). Importantly, other parameters of performances (e.g., ***Q***, FWHM, FSR, peak intensities) were fairly steady during tuning procedures, well showing the practical potential of the protein-based 3D WGM microlasers here. Along with increasing involvement in bio-related edge-cutting fields like label-free high-sensitivity detection^18^,^19^,^20^,^21^,^22^ and optical micromanipulation^36^, protein-based 3D WGM microlasers might open new opportunities for biological and medical applications such as optical biosensing, diagnosis, tunable “smart” biophotonics.

## Results and Discussion

### Preparation of BSA/RhB composite aqueous ink

**Figure 1:**
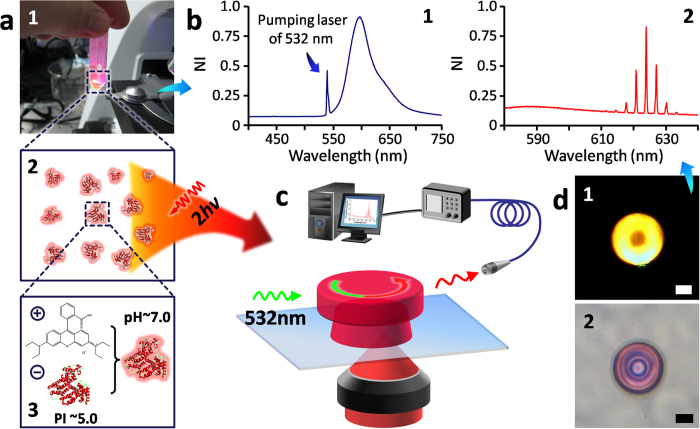
Schematic of FsLDW-fabrication of protein-based 3D WGM microlasers and related tests of lasing actions. (**a**) 1, BSA/RhB composite aqueous ink for FsLDW; 2 and 3, illustration of BSA biomacromolecules loaded with RhB small molecules via electrostatic force in neutral aqueous solutions. (**b**) 1, fluorescent spectrum of BSA/RhB composite aqueous ink under 532-nm laser light excitation; 2, lasing spectrum from a proten-based 3D microdisk WGM-laser with diameter of 30 μm. (**c**) The schematic of aqueous FsLDW and lasing test of protein-based 3D WGM microlaser. (**d**) 1, the dark-field fluorescence microscopy image of the 30-μm-diameter proten-based 3D microdisk WGM-laser in 2; 2, optical microscopic image of a 30-μm-diameter proten-based 3D microdisk WGM-laser; scale bar, 10 μm. The schematic was drawn by Yun-Lu Sun.

When dissolved in water, RhB molecules would discretely and site-selectively bind onto BSA biomacromolecules at so-called site I and site II which are located in hydrophobic cavities in BSA sub-domains IIA and IIIA, respectively^37^. As illustrated in Fig. 1a, [RhB]^+^ is electropositive, and BSA (isoelectric point, PI, ~4.7) is electronegative in pure water. According to work by Ji-Ye Cai *et al.*^37^, when the molar ratio of RhB to BSA in solution was <50, RhB bound with BSA at site I by hydrophobic and electrostatic interactions. Yet if the molar ratio (RhB: BSA) was more than 50, binding at site II started. The RhB combination at site I had higher binding affinity to be of great help for obtaining stable BSA/RhB composite protogels. During protein-FsLDW in our work, BSA concentration should be relatively high usually of ~100–1000 mg/mL as needed. So, even if the RhB concentration reached its solubility in water, namely, ~15 mg/mL, the RhB: BSA molar ratio was still much smaller than 50 here. As a result, the binding was dominated by site-I type. It ensured the fairly steady loading of RhB in BSA molecule structure and, further, the protein hydrogel. Meanwhile, this discrete binding of RhB onto specific sites of BSA biomacromolecules might effectively avoid high-concentration-induced fluorescence quenching of dimerized RhB^38^. It was helpful for loading more RhB without RhB dimerization, and increasing fluorescent intensity. These points are quite vital and beneficial prerequisites for operation stability and good performances of subsequent protein-based microlasers, especially, in aqueous environments.

### FsLDW-fabricated protein fluorescent micro/nano-hydrogels

Two photochemical issues might facilitate the high-degree photocrosslinking of BSA molecules during FsLDW here: BSA “self-sensitized” photocrosslinking^30^,^31^ and probable RhB photosensitization^32^,^33^,^34^,^35^. In detail, on one hand, some amino-acid residues in protein molecules (e.g., BSA here), for instance, tryptophan (Trp), have been proved to be able to generate singlet oxygen (^1^O_2_) under light irradiation^30^. It means that BSA photocrosslinking might occur in solutions even without any specific photosensitizers added. Based on this phenomenon, protein-based laser micro/nano-fabrication has been successfully realized by using pure protein inks (without specific photosensitizers added)^31^. According to previous reports of Prof. Jason B. Shear’s group, the protein concentration in pure protein aqueous solution needs to be high enough to ensure sufficient reactive species supplied by protein’s “self-photosensitization” (for example, ~200–400 mg/mL for BSA)^31^. In this work, much higher BSA concentration (up to ~800–1000 mg/mL) was applied so that nearly saturated BSA aqueous solution was obtained with high viscosity for better 3D FsLDW. It meanwhile contributes to the high-degree “self-photosensitized” photocrosslinking of BSA and therefore high quality of well-customized 3D BSA-based micro/nano-structures/devices. On the other hand, though the yields are relatively low by using RhB as the two-photon photosensitizer, high-concentration RhB might still help to produce reactive species under high-power-intensity laser irradiation (like ^1^O_2_^32^, ^33^, ^34^, superoxide (O_2_∙^−^)^35^, and even some other not-yet-identified oxidative species probably^35^). Then, these reactive species may induce redox polymerization and crosslinking of residues of protein molecules (e.g., His, Tyr, Trp, Cys, Met)^3^. So, BSA photocrosslinking might be obviously promoted especially when the RhB concentration is so high (5 mg/mL here). Thus, the above two key factors might function simultaneously to enable the high-degree photocrosslinking of BSA and then high-quality FsLDW customization (both surface and 3D geometry) of protein-based optical micro/nano-devices.

**Figure 2:**
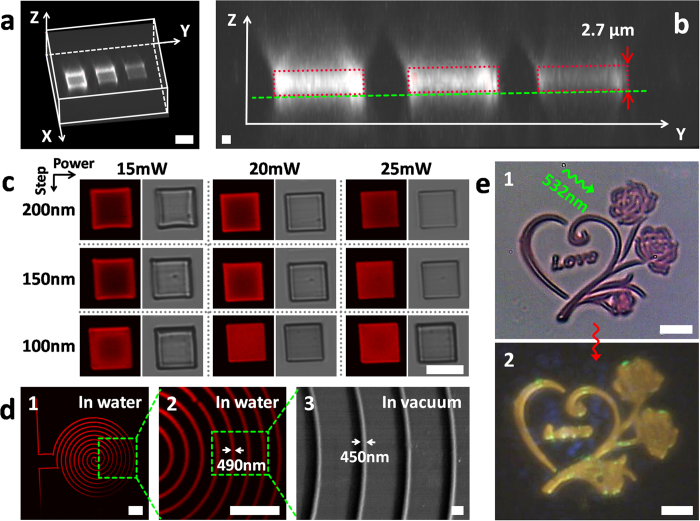
Optimization of 2D-FsLDW and characterization of FsLDW-fabricated BSA/RhB hydrogel micro/nano-structures by confocal microscopy, SEM and optical microscopy (OM). (**a**) The 532-nm-excited 3D-reconstructed confocal microscopic fluorescent image of three BSA/RhB hydrogel micro-squares with different FsLDW scanning steps. Scale bar, 10 μm. (**b**) Y-Z-plane cross-section confocal microscopic fluorescent image of (**a**). Scale bar, 1 μm. (**c**) Confocal microscopic fluorescent (left in each column) and bright-field (right in each column) top-view images of BSA/RhB micro-squares in air FsLDW-fabricated with different scanning step and laser power. Scale bar, 10 μm. (**d**) Confocal microscopic fluorescent images in water (1 and 2, scale bar, 10 μm) and SEM image in vacuum of a BSA/RhB hydrogel spiral nano-line (3, scale bar, 1 μm). (**e**) 1, optical microscopic image of a BSA/RhB hydrogel relief micro-sculpture of roses; 2, fluorescent images of “golden” roses in 1 after filtering sub-565-nm light including 532-nm excitation light; scale bar, 10 μm.

As shown in Fig. 2, fluorescent protein-hydrogel-based micro/nano-structures were obtained by FsLDW with a variety of processing parameters. Figure 2a exhibits confocal microscopy 3D reconstructed image of three BSA/RhB micro-squares excited by 532-nm light. The three BSA/RhB micro-squares in Fig. 2a,b were FsLDW-fabricated with different scanning steps (from left to right in Fig. 2a,b: 100 nm, 150 nm, and 200 nm, respectively; laser power, 20 mW; exposure time on single point, 1000 μs). Figure 2b is the Y-Z-plane cross-section image of Fig. 2a. In Fig. 2b, the distribution of fluorescence intensity observed from the side indicated preliminarily that fluorescent RhB was uniformly loaded inside BSA hydrogels. Additionally, probably because of negative correlation of hydrogel-crosslinking density to FsLDW scanning step, it is obvious in Fig. 2a,b that the fluorescent intensity of BSA/RhB composite hydrogels (positively correlated to density of RhB) increased along with the reduction of scanning step. Therefore, one facile method was achieved here to adjust content and therefore density of RhB in BSA hydrogels as needed by changing FsLDW scanning step.

In accordance to our previous work, the exposure time on single point was chosen to be 1000 μs for aqueous protein-FsLDW to ensure sufficient photocrosslinking of protein molecules^3^. Further in Fig. 2c, by confocal microscopy characterization in air, top-view fluorescent (left in each column) and bright-field (right in each column) images showed fluorescence distribution and geometry quality of BSA/RhB micro-squares fabricated with different two other key processing parameters (scanning step and laser power). The darker areas in some micro-squares might be caused by two matters: 1, part of top surface was out of focal plane for shrinkage and deformation of BSA/RhB hydrogels; 2, less BSA bound with RhB was polymerized inside hydrogel. Both of the two points were probably related to lower hydrogel-network crosslink density caused by smaller laser power intensity or larger FsLDW scanning step. So, several parameter sets (1000-μs/100-nm/20-mW, 1000-μs/100-nm/25-mW, and 1000-μs/150-nm/25-mW) could lead to high FsLDW quality indicated by uniform fluorescence and well-defined geometry in Fig. 2c. However, the BSA micro-square fabricated with 100-nm scanning step and 25-mW laser power was kind of “bloated” for excessive degree of photo-polymerization. Meanwhile, time consuming of direct-writing process, as a technical shortcoming of FsLDW, needs to be reduced for higher fabrication efficiency, especially for relatively big 3D microstructures. Since scanning steps were on three dimensions, reasonably increased scanning steps can efficiently shorten be FsLDW-processing time. By adopting 150 nm instead of 100 nm, processing time would be reduced to only (100/150)^3^ = 8/27 of original one. But resulted bigger voxel distance needs to be offset by higher laser power for maintaining originally high fabrication quality. Thus, BSA/RhB micro/nano-devices would be fabricated with 150-nm scanning step and 25-mW laser power for our self-made FsLDW system via comprehensively consideration of processing quality (surface morphology and 3D geometry) and consumed time of FsLDW.

With this preliminary optimization of protein-FsLDW, hyperfine 2D and 2.5D micro/nano-structures could be directly written out from protein aqueous ink (see Fig. 2d,e) to demonstrate its availability for nanoprocessing. An as-fabricated high-quality spiral nano-line showed width of ~490 nm after equilibrium swelling in water (Fig. 2d1,2) and ~450 nm in vacuum (Fig. 2d 3). Additionally, BSA/RhB-hydrogel relief micro-sculpture of roses was well constructed with more structure-details (Fig. 2e 1), and looked like interesting “golden” roses in Fig. 2e 2 by filtering sub-565-nm light including 532-nm pumping laser for clear fluorescent images (see Figure S1 in Supplementary Information (SI), the same for other fluorescent images in this paper). In conclusion, the protein-based optical active material was obtained here with satisfactory multiphoton photo-processability.

### True 3D aqueous-FsLDW of protein-based 3D WGM microlasers with improved protein reagent and scanning mode

Compared with previously reported FsLDW in hard materials like silica^23^ and solid resins^39^,^40^,^41^ ,true 3D processing and forming were of more challenges to conquer, particularly, for soft matters' direct-write additive manufacturing techniques including FsLDW^42^, inject writing^43^, and 3D printing^44^. That is, solid matrixes 3D-surrounding scanned areas were needed to sufficiently support overhanging, even suspended and unconstrained “true-3D” parts of soft structures fabricated^42^,^43^,^44^. Whereas, the true-3D parts of soft structures would probably deform, drift, and collapse without sufficient support during simple layered scanning^42^. Thus, as one useful and important way of improvement for protein true-3D aqueous-FsLDW, high-viscosity of aqueous protein ink was prepared by controlled evaporation of water to provide enough support in the work of Eric C. Spivey *et al.*^42^. But it might have limitations to consider only the viscosity of ink. On one hand, over-high concentration and viscosity of water-evaporated protein “protogels” might cause inconvenience to *in situ* processing in microfluidic chips or in environments with live cells, and bring negative influence on applicability. On the other hand and more importantly, the morphology (especially surface) quality of protein-based true 3D devices might be not good enough for optical applications^42^. Although other improvements for true-3D aqueous-FsLDW were developed for instance of “dynamic biomimetic laser-fabrication” based on 3D wrinkling^45^, it was relatively short of satisfactory designability, controllability and material-diversity.

**Figure 3:**
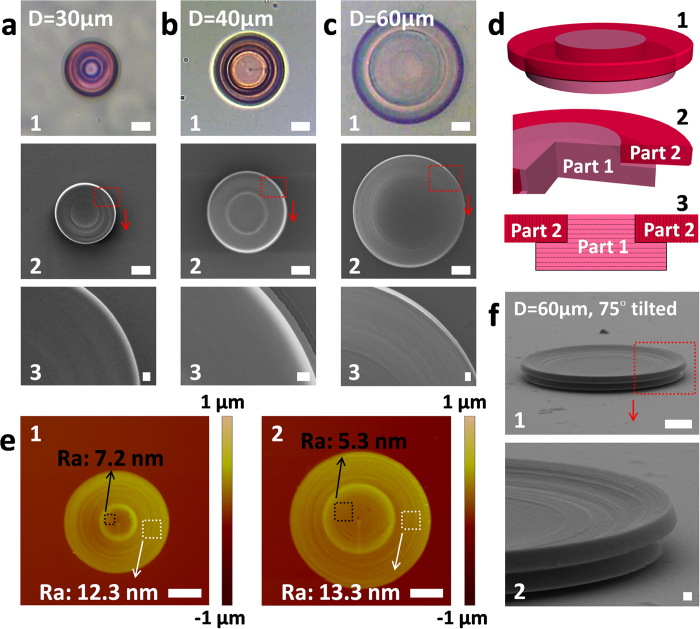
Characterization of optimized-FsLDW-fabricated protein-based true 3D WGM microlasers (2.5-μm designed thickness of upper-part microdisks) by OM, SEM and AFM. (**a**) The protein-based 3D WGM microdisk with diameter of 30 μm. 1, top-view OM image; 2, top-view SEM image; 3, SEM partial enlarged detail of image 2. Scale bars, 1 and 2, 10 μm; 3, 1 μm. (**b**) The protein-based 3D WGM microdisk with diameter of 40 μm. 1, top-view OM image; 2, top-view SEM image; 3, SEM partial enlarged detail of image 2. Scale bars, 1 and 2, 10 μm; 3, 1 μm. (**c**) The protein-based 3D WGM microdisk with diameter of 60 μm. 1, top-view OM image; 2, top-view SEM image; 3, SEM partial enlarged detail of image 2. Scale bars, 1 and 2, 10 μm; 3, 1 μm. (**d**) Schematic illusration of true 3D WGM microdisk FsLDW-fabricated with partly conformal scanning mode. (**e**) AFM characterization of protein-based microdisks (1, 30-μm diameter; 2, 40-μm diameter) without bottums. Scale bar, 10 μm. (f) 1, SEM image of a 75° tilted protein-based true-3D microdisk with a 60-μm diameter, scale bar, 10 μm; 2, SEM enlarged view of image 1, scale bar, 1 μm.

Otherwise, “conformal” trajectory of writing (mode of scanning) was another facile and effective factor for realizing true-3D ability of direct-write techniques^46^,^47^. This has been well regarded and widely utilized in 3D-printing technologies^46^,^47^. But it has not been emphasized in FsLDW, for which simply layered scanning was used usually (see **Video 1**), as far as we know. Herein, we introduced partly conformal scanning into protein aqueous-FsLDW, and properly increased protein concentration and viscosity of ink simultaneously. So, true-3D aqueous FsLDW of protein was ensured with high quality (see Fig. 3a–c) for optical application. BSA concentration in FsLDW ink was as high as 800–1000 mg/mL for adequate viscosity (RhB, 5 mg/mL). On the other hand, as shown in Fig. 3d and animation of FsLDW (**Video 2**), protein 3D WGM microlasers were divided into two parts: 1, the base with parallel-layered scanning (pink Part 1); 2, the overhanging outer ring with vertical-layered scanning (rosy Part 2). That is, conformal scanning was applied in Part 2 of overhanging outer ring so that the structure in fabrication made itself the supporting matrix during additive FsLDW micro/nano-manufacturing. Based on the preliminary optimization of 2D protein FsLDW, optimal parameters were adopted for protein 3D WGM microlasers as shown in Fig. 3 (scanning step, 150 nm; laser power, 25–28 mW; exposure time on single point, 1000 μs). By atomic force microscopy (AFM) characterization in Fig. 3e, the device surfaces parallel to scanning layers trended to have much lower average roughness (Ra) as low as ~5 nm (see **Experimental Section**). It guaranteed the smoothness of microdisk side surface (see Fig. 3f 2) for WGM optical resonating and lasing, which greatly helped to improve reflection and to obtain high ***Q***.

Additionally, the combination of these FsLDW parameters was experimentally demonstrated to be appropriate to true 3D fabrication of protein-based WGM microlasers, especially for overhanging parts (Fig. 3f and Figure S2a in SI). Besides, the high failure rate of FsLDW with simple layered scanning proved the necessity of conformal scanning for aqueous true 3D FsLDW here (see Figure S2 g, h, and i).

### Lasing performances of protein-based 3D WGM active microcavities in air

**Figure 4:**
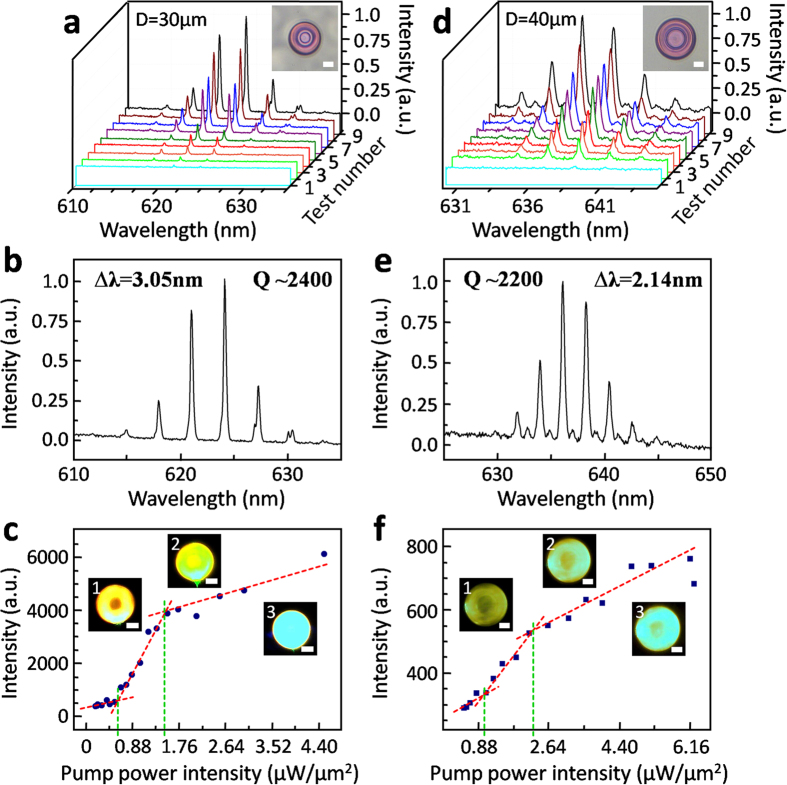
PL spectra analysis of protein-based 3D WGM microlasers in air (2.5-μm designed thickness of upper-part microdisks). (**a**) 3D-waterfall arranged PL spectra from a 30-μm-diameter protein-based 3D WGM microlaser (see inset optical microscopic image, scale bar, 10 μm) pumped with increasing intensities of 532-nm ps-laser light. (**b**) The spectrum of test number 9 in (**a**) pumped by ~1.03-μW/μm^2^ 532-nm ps-laser light. (**c**) Peak values of PL spectra from the protein-based 3D WGM microlaser in (**a**) *vs* 532-nm pumping intensities. Insets: optical microscopic fluorescent images of the protein-based 3D WGM microlaser in (**a**) under different pumping intensities; 1, ~0.44 μW/μm^2^; 2, ~1.32 μW/μm^2^; 3, ~2.2 μW/μm^2^; scale bar, 10 μm. (**d**) 3D-waterfall arranged PL spectra from a 40-μm-diameter protein-based 3D WGM microlaser (see inset optical microscopic image, scale bar, 10 μm) pumped with increasing intensities of 532-nm ps-laser light. (**e**) The spectrum of test number 9 in (**d**) pumped by ~3.97-μW/μm^2^ 532-nm ps-laser light. (**f**) Peak values of PL spectra from the protein-based 3D WGM microlaser in (**d**) *vs* 532-nm pumping intensities. Insets: optical microscopic fluorescent images of the protein-based 3D WGM microlaser in (**d**) under different pumping intensities; 1, ~0.58 μW/μm^2^; 2, ~1.48 μW/μm^2^; 3, ~2.64 μW/μm^2^; scale bar, 10 μm.

Owing to our efforts on optimization of FsLDW (including ink viscosity, scanning mode, and processing parameters), high-quality protein-based 3D microdisks were directly written out. Then, under proper excitation of pumping light from a 532-nm picosecond laser (see **Experimental Section**), fluorescence of loaded RhB recirculated and enhanced along the side surface of a protein-based 3D microdisk (so-called WGM resonation) to lead to lasing^29^, as schematically illustrated in Fig. 1c. Photoluminescence (PL) spectra in air were exhibited and analyzed in Fig. 4, proving excellent operation of protein-based 3D WGM microlasers. Here, side surface with nano-scale roughness (~ 5 nm, see Fig. 3e,f-2, and Figure S2 c and f) resulted into the scattering and leakage of light WGM-cycled in protein-based 3D microdisks. And spectra were remotely collected by a fiber-optical spectrometer (Fig. 1).

According to intensity increasing of pumping light (see Figure S3 in SI for light power intensity estimation and **Experimental Section**), lasing of a protein-based 3D WGM microlaser with 30-μm diameter (***D***) was started and enhanced as proved in Fig. 4a–c. And similarly, PL of a 40-μm-***D*** protein-based 3D WGM microlaser was depicted in Fig. 4d–f. Several PL spectra 3D-waterfall-arranged in Fig. 4a rose along with increasing intensity of pumping laser until that sharp peaks started to emerge from the emission spectrum around ~618–630 nm (so-called lasing action), when pumping intensity exceeded the threshold (***I***_th_, ~ 0.57 μW/μm^2^ for the device in Fig. 4a). Peak values of PL spectra from the protein-based 3D WGM microlaser in Fig. 4a under different pumping intensities were dotted in Fig. 4c. As shown in Fig. 4a,c, after spontaneous fluorescence started to be turned into stimulated emission (lasing) under ~0.57 μW/μm^2^ pumping by WGM oscillation, lasing intensity of the protein-based 3D WGM microlaser increased linearly. Till pumping intensity was ~1.56 μW/μm^2^ (~2.7***I***_th_), the output lasing intensity began to become approximatively saturated, which might be caused by gain saturation and dye photobleaching under repeated overhigh exposure to pumping laser (Fig. 4c). Similarly, for a 40-μm-***D*** protein-based 3D WGM microlaser in Fig. 4d, fluorescence was WGM-stimulated into lasing and linearly enhanced from pumping intensity***I***_th_ of ~0.88 μW/μm^2^, and was saturated till ~2.16 μW/μm^2^ pumping (Fig. 4f). Additionally, the inset images in Fig. 4c,f were obtained by a self-made dark-field fluorescence microscope integrated in the testing system, and visually showed the PL and lasing actions of protein-based 3D WGM microlasers. Here, existence of pumping thresholds, and further nonlinear PL increase versus pumping intensity, were exhibited as fingerprint indicating lasing phenomenon of protein-based 3D WGM microlasers.

Further, for an isolated circular WGM microdisk, ***Q*** value could be determined by formula as follow^27^,^28^,
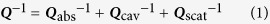
Where ***Q***_abs_^−1^, ***Q***_cav_^−1^, and ***Q***_scat_^−1^ corresponded respectively to absorption loss, cavity finesse, and scattering loss. Since BSA protein hydrogel and RhB were transparent for visible light (especially around 620 ~ 630-nm wavelength, see Figure S4 in SI), absorption loss and therefore***Q***_abs_^−1^ was less important. And ***Q***_cav_^−1^ might be much smaller than***Q***_scat_^−1^. Consequently, scattering loss was probably the major factor influencing total ***Q*** here, and
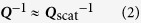
So,***Q***value of protein-based 3D WGM micro-devices here was sensitively influenced by quality of surface and 3D shape which were well-tailored by FsLDW. Further, ***Q***value could be estimated according to the spectral parameters by formula of
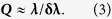
Here, ***λ*** is the resonance wavelength, and***δλ*** = FWHM.

In detail, the spectrum of test number 9 in Fig. 4a was pumped by ~1.03 μW/μm^2^ 532-nm ps-laser light (see Fig. 4b). For the spectrum in Fig. 4b, FWHM (***δλ***) was ~0.26 nm at wavelength of ~624.1 nm (the highest peak probably resulted from mode competition). And ***Q*** value was calculated to be ~2400 with formula (3). Similarly, lasing spectra of a 40-μm-diameter protein-based 3D WGM microlaser were exhibited in Fig. 4d. For the spectrum of test number 9 in Fig. 4d (see Fig. 4e), FWHM was ~0.29 nm at wavelength (***λ***) of ~638.6 nm (the highest peak), and ***Q*** was estimated to be ~2200. In our work, ***Q*** of protein-based 3D WGM microlasers was much higher than previously reported WGM microlasers made of traditional artificial polymers without annealing post-processing^27^, and was similar to those based on organic crystals with much higher refractive indexes (RI)^28^. It might be caused by high-quality morphology (Ra, ~5 nm) due to FsLDW-fabricated protein hydrogels’ efficient “self-smoothing” phenomenon^3^. Although there was no secondary annealing to avoid excessive damage to biomacromolecules and adverse impact on bio-applications, protein-based 3D WGM microlasers were still endowed with higher ***Q*** by nano-scale excellent quality control of surface and 3D geometry during protein-FsLDW (see Fig. 2 and Fig. 3). And the nano-scale roughness of side surface of protein-based 3D microdisks (see Fig. 3e,f-2, and Figure S2 c and f) enabled scattering and leakage of WGM-cycled fluorescence. So the PL spectra could be remotely collected for analysis. Theoretically, if annealing processing was applied as frequently used in other WGM-related reports, much higher ***Q***might be able to be achieved^18^,^23^.

Moreover, for the 30-μm-diameter protein-based 3D WGM microlaser in Fig. 4a, the free spectral range (FSR, ***Δλ***) was ~3.05 nm at ~618–630 nm (Fig. 4b). And for 40-μm diameter in Fig. 4d, FSR was ~2.14 nm at ~632–643 nm (Fig. 4e). Thus, RI of protein hydrogel (***n***_hydrogel_) here was estimated to be ~1.47 in air using formula as follow^27^,
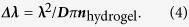
***n***_hydrogel_ of FsLDW-fabricated BSA/RhB hydrogels here was found to be smaller than BSA/MB (methylene blue) hydrogels in our previous work (see Figure S5 in SI)^3^,^4^,^5^.

### Lasing performance of protein-based 3D WGM active microcavities in aqueous environment

**Figure 5:**
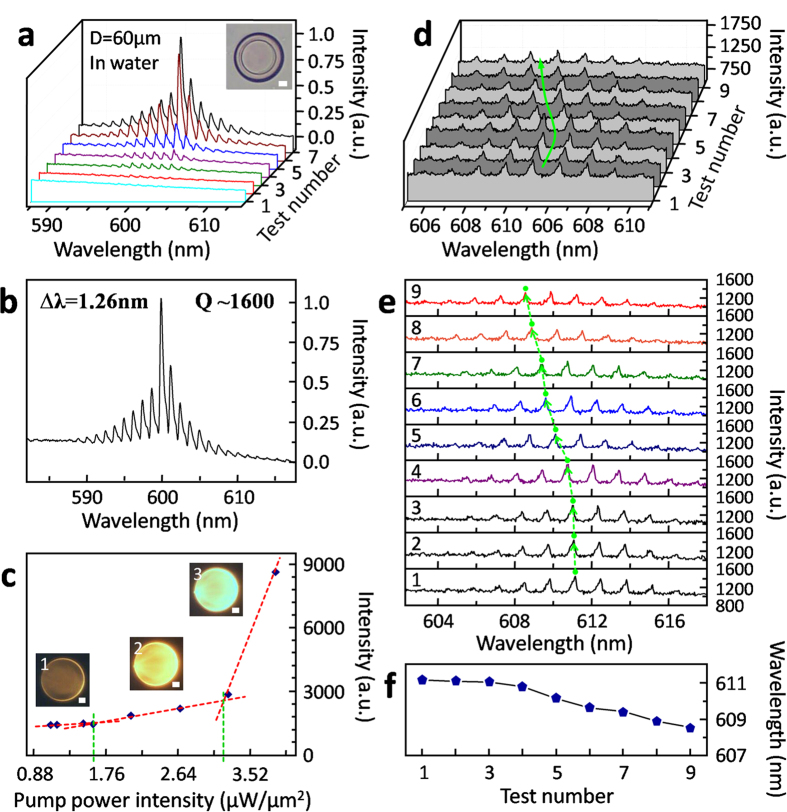
PL spectra analysis and adjustable lasing action of a protein-based 3D WGM microlaser in aqueous environments. (**a**) 3D-waterfall arranged PL spectra from a 60-μm-diameter (2.5-μm designed thickness of upper-part microdisks) protein-based 3D WGM microlaser in pure water (see inset optical microscopic image, scale bar, 10 μm) pumped with increasing intensities of 532-nm ps-laser light. (**b**) The spectrum of test number 7 in (**a**) pumped by ~3.84-μW/μm^2^ 532-nm ps-laser light. (**c**) Peak values of PL spectra from the protein-based 3D WGM microlaser in (**a**) *vs* 532-nm pumping intensities. Insets: optical microscopic fluorescent images of the protein-based 3D WGM microlaser in (**a**) under different pumping intensities; 1, ~1.17 μW/μm^2^; 2, ~2.07 μW/μm^2^; 3, ~3.26 μW/μm^2^; scale bar, 10 μm. (**d**) 3D-waterfall arranged lasing spectra from a 60-μm-diameter (2.5-μm designed thickness of upper-part microdisks) protein-based 3D WGM microlaser in aqueous solutions with increasing Na_2_SO_4_ concentrations; pumping intensity of 532-nm ps-laser light, ~2.0 μW/μm^2^. (**e**) 2D stacked lasing spectra in (**d**). (**f**) Wavelength blueshift of a particular peak in lasing spectra (near to central wavelength) of (a) and (**b**) along with Na_2_SO_4_-concentration increasing.

Even in aqueous environments, WGM lasing ran well for protein-based 3D WGM active microcavities with 60-μm or larger diameter (see Fig. 5a–c). Similarly to those in air, several PL spectra of a 60-μm-diameter protein-based 3D WGM microlaser in pure water (inset of Fig. 5a) were 3D-waterfall arranged in Fig. 5a for visually exhibition of rising peaks (around 600 nm) according to increasing pumping intensity (see **Experimental Section**). Here, ***I***_th_ of pumping laser was ~1.61 μW/μm^2^ for the WGM microdevice in Fig. 5a to start lasing in water (see Fig. 5a,c), higher than that in air. After linear increasing of lasing peaks from ~1.61 μW/μm^2^ to ~3.17 μW/μm^2^, slope of lasing spectra's increasement was drastically enlarged by high pumping laser over ~3.17 μW/μm^2^ (Fig. 5c). However, during the tests under overhigh pumping laser power intensity (~3.97 μW/μm^2^), lasing spectra from the WGM microlaser in Fig. 5a tended to collapse (probably resulted from gain saturation, dye photobleaching and even pumping laser ablation). So, lasing spectra under overhigh pumping intensities were not explored further and exhibited here. What’s more in Fig. 5c, lasing actions of the protein-based 3D WGM microlaser in water were visually displayed by the inset dark-field fluorescence microscopic images. For detailed analysis, number-7 spectrum in Fig. 5a pumped with ~3.83 μW/μm^2^ 532-nm light was shown in Fig. 4b. In Fig. 4b, δλ was ~0.38 nm for the peak at ~600 nm. So, ***Q*** was ~1579 calculated by formula (3). Also, for other WGM lasing tests in Fig. 5d–f, ***Q*** was relatively large around ~2000–~3300 for a 60-μm-diameter protein-based 3D WGM microlaser in aqueous environments (see Figure S9).

Significantly, lasing ability in aqueous environments was harder to be achieved for WGM microresonators based on polymers and other organic materials^28^. There was few related reports to our knowledge except the only case of initial implementation by high-RI organic crystals^28^. Let alone their further valuable utilizations in aqueous environments. In theory, WGM optical resonation was realized in microdisks by trapping light via total internal reflection at circular boundary between dielectric cavities and surroundings. The reflectivity at the side-surface boundary would decrease along with increase of surrounding medium RI and result smaller RI difference between WGM cavities and surroundings. As a result, it might reduce ***Q***and confinement factor (***F***), and make the WGM-lasing threshold extremely high. And even the lasing resonation would disappear. Specially for the situation here, besides the higher RI of aqueous solutions, the equilibrium swelling phenomenon in aqueous solutions would induce reduction of RI of protein hydrogels^3^,^4^. Thus, the confinement of light in WGM optical cavities should have decreased more obviously, leading to much smaller ***F*** and ***Q***. But, on the contrary to expectation, there was no indication that ***Q*** reduced during operation in aqueous solutions. This might be precisely due to hydrogel’s equilibrium swelling (like a double-edged sword) in water that smoothened nano-wrinkles of side face of WGM optical cavities. This stable light-confinement performance in aqueous environments indicated their successful potential bio-related utilizations like water-phase biosensing.

Because of equilibrium swelling, the diameter of a protein microdisk in aqueous solutions would be a little larger than that in air^3^,^4^,^15^,^48^, as proved in Figure S6. Therefore, the real diameter of the 60-μm-diameter protein-based 3D WGM microlasers in water in Fig. 5 was ~65 μm (Figure S6), which was in good agreement with Fig. 2d. And FSR (***Δλ***) was ~1.26 nm at ~600 nm in Fig. 5b; *Δλ* was ~1.31 nm at ~611 nm in Fig. 5e1, 2, 3. So, by formula (4), average effective RI was estimated to be ~1.40 for FsLDW-fabricated BSA/RhB hydrogels in water (approximately equal to absolute RI here). Additionally, since relative low RI of protein hydrogels might cause significant leakage of confined light at the interface between devices and substrates (glass coverslips here, RI ~1.6), true 3D geometry of protein WGM microlasers became more important and necessary for WGM optical confinement and oscillation, especially in aqueous environments. Particularly, the optical features of FsLDW-fabricated protein-based hydrogels, including RI, transmission (absorption) property and fluorescence, were systematically explored in this work. Together with our previous reports^3^,^4^,^5^,^11^, it was significant for applications of novel protein-based optical materials, and helped to practically provide this new option of biomacromolecule-based biopolymer and hydrogel to photonic material library.

### Stimulus-responsively adjustable lasing actions of protein-based 3D WGM active microcavities in aqueous environment

Furthermore, stimulus-responsive adjustment of lasing actions in aqueous environments was demonstrated and studied using a protein-based 3D WGM microlaser by merely changing Na_2_SO_4_ concentration of aqueous solution (see Fig. 5d–f). This protein-based 3D WGM microlaser had a designed diameter of 60 μm but a real one of ~65 μm in pure water for equilibrium swelling (see Figure S6). As proved in Fig. 4a,d and Fig. 5a, protein-based 3D WGM microlasers worked well during continuously multiple exposures by 532-nm laser light even with increasing power intensities. Here, to reduce possible impact from dye photobleaching or even laser ablation, moderate pumping laser intensity was fixed to be ~2.0 μW/μm^2^ for repeated 532-nm laser exposures and exsited lasing actions during stimulus-responsive adjustments in Fig. 5d–f.

The central wavelength was the major factor to be tuned here as visually revealed in Fig. 5d, where central wavelength was blue shifted regularly along with increasing Na_2_SO_4_ concentrations. According to Figure S7 and Figure S8, the central wavelengths corresponding to highest peaks of lasing spectra were nearly constant during multiple lasing tests except some accidental saltations probably because of mode competition. And the position of a certain lasing peak was approximately constant during multiple exposures. It proved the stable lasing of protein-based 3D WGM microlasers if not-overhigh and unchanged pumping intensities were utilized. For further discussion, the adjusted lasing spectra were 2D stacked in Fig. 5e. The nearly identical three spectra of number 1, 2, and 3 in Fig. 5d,e were collected successionally from the protein-based 3D WGM microlaser immersed in pure water, proving the negligible influence from other factors like repeated pumping-laser exposures. Then, for spectra from 3 to 9, Na_2_SO_4_ concentration was gradually increased with the step of 8.33 × 10^−4^ mol/L (see Table S1 of SI) and no any other change in the total system. During this process, the central wavelength of lasing spectra went down nearly linearly step by step from ~611 nm to ~608.5 nm (spectrum 3–9) in Fig. 5f and Table S1. In addition, a series of detailed data were listed in Table S1. Other main performances were found to stay approximately steady from data in Table S1 and Figure S9 in SI. In Figure S9a, the peak intensity of the lasing spectra during adjustment slightly fluctuated around ~1327–1526 without obvious trend of increase and decrease. FSR also stayed at around ~1.31 steadily (Figure S9b). And ***Q*** was around ~2000–3300 (except a accidental datum point of ~1488) without obvious regular trend of change (Figure S9c) in correspondence with relatively stable FWHM around ~0.2 nm (Figure S9d). In conclusion, we facilely achieved the stimulus-responsive “smart” adjustment of central wavelength of lasing of a protein-based 3D WGM microlaser in aqueous environments, and simultaneously kept other main performance parameters fairly stable.

As well proved and applied in previous reports^48^, the equilibrium-swollen state of FsLDW-fabricated protein hydrogels inherently and sensitively responds to changes of ionic strength in aqueous surroundings (e.g., changed Na_2_SO_4_ concentrations). Na_2_SO_4_ is a non-chaotropic salt like NaCl, Na_2_HPO_4_, and NaH_2_PO_4_ that do not directly act upon proteins. Implied by the “salting out” phenomenon of sulfate on dissolved BSA, Na_2_SO_4_ was chosen here for demonstration because it might be more effective at contracting swollen BSA hydrogels and tuning lasing action of a protein-based 3D WGM microlaser^48^. In aqueous solutions, it might merely induce slight contration of the protein-based 3D WGM microlaser by slowly increasing Na_2_SO_4_ concentration with the step of 8.33 × 10^−4^ mol/L^48^. It was difficult to be directly distinguished under an optical microscope. But the ~611-nm-to-608.5-nm blueshift of lasing spectra might reflect the Na_2_SO_4_-concentration-change-induced slight shrinkage of the protein-based 3D WGM microlaser. In correspondance with hydrogel shrinkage and water extrusion out, the effective RI ***n***_eff_ (approximately equal to ***n***_hydrogel_ here) of protein hydrogel should increase, while the diameter of the protein-based 3D WGM microlaser should decrease. For formula (4), it should be noted that FSR (***Δλ***) was tested to be steady at around ~1.31 in Figure S9b, and ***λ***buleshifted regularly with increasing Na_2_SO_4_ concentration. Theoretically, product ***D*****∙*****n***_hydrogel_ was supposed to get smaller as a result. It meant that decrease of ***D*** caused by shrinkage, though indistinguishable for such slight Na_2_SO_4_-concentration change, still played a dominant role in tuning lasing actions here.

### Advantages and potential applications of the FsLDW-fabricated protein-based micro/nano-devices

Traditional FsLDW monomers are mainly artificial synthetics for example of epoxy resins (e.g., SU8) and acrylate-based monomers (e.g., poly(ethylene glycol) diacrylate (PEGDA))^39^. Compared with them, some apparent advantages might be comprehensively achieved for the protein-based 3D WGM microlasers by using FsLDW-processable protein-based/-derived biopolymers (e.g., BSA, avidin, enzymes^3^,^4^,^5^,^14^,^15^,^48^): **(i)** Excellent biocompatibility of FsLDW-fabricated protein-based micro/nano-hydrogels (even including FsLDW-processing environment) has been well proved by series of work published previously (e.g., live bacteria/cells guiding and patterning^15^, micro-niche culturing^48^, motile-bacteria-actuated micromechanics^42^, “*in situ*” manipulation of neuronal development^15^). Besides, in our previous work, FsLDW-fabricated protein-based optical microdevices (like microlenses) were experimentally demonstrated of satisfactory antibiofueling performance^3^, indicating the good biocompatibility as well. **(ii)** Eco-friendliness would be better for replenishable natural resources^1^ and biodegredation^5^ of protein-based biopolymers, as well as aqueous FsLDW-processing without organic solvents^3^,^4^,^5^. **(iii)** Some intrinsic functions of proteins might be at least partly maintained after FsLDW processing to enable various inherent features (difficult to replicate via artificial materials), such as biocatalysis^14^, specific recognition^11^,^15^, cell adhesion^49^, optical transparency^2^,^6^,^7^,^8^,^9^, “smart” environmental responsiveness^3^,^4^, bio-illumination^8^,^10^, tailorable soft mechanical characteristics^2^,^5^,^6^,^8^. **(iv)** Protein-based/-derived FsLDW biomaterials could be facilely and flexibly functionalized with methods like blending^16^,^17^, copolymerization during FsLDW^11^, and chemical modification and loading^16^,^17^. All these merits are of great help for wide utilizations of FsLDW-fabricated protein-based photonics including the 3D protein-based WGM microlasers obtained in this work.

In our experiment, lasing actions of the 3D protein-based WGM microlasers were tuned by changing ionic strengths. The ionic-strength-based approach is actually an important technical option for research and application of “smart” hydrogels including FsLDW-fabricated protein-based micro/nano-hydrogels^48^, for example, ionic-strength-actuated valves, pumps, clutches, and optics. Since all live cells, tissues, and organs need appropriate environments to keep normal vital movements, proper ionic strengths are precisely one of the most important essential requirements^50^,^51^. On the other hand, the change of ionic strengths might be the presentation of physiological activities (e.g., ionic strength changes *in vivo*^50^ or caused by fermentation processes^51^). Meanwhile, compared with other mechanisms, especially like responsiveness via changing organic solvents, much better eco-/bio-compatibility and operation convenience might be brought by the aqueous ionic-strength-based method. Therefore, it might have great potential for bio-related sensing and self-adaptive control to trigger or actuate actions of “smart” device and systems via ionic strength responses (e.g., the ionic-strength-responsive lasing actions of BSA-based 3D WGM microlasers).

## Conclusion

In summary, for the first time as far as we konw, proteins (BSA here), a natural-biomacromolecule-based novel optical material, were utilized as biocompatible and multifunctional host material to FsLDW-fabricate 3D WGM microlasers. RhB, site-selectively and discretely loaded onto BSA biomacromolecules, first acted as a photonsensitizer in aqueous ink for FsLDW and then worked well as the luminescent component. As-formed BSA/RhB composite hydrogel was fully proved to be an ideal photo-machinable biopolymer and an innovative optical active medium by successful FsLDW-fabrication of series of delicate protein fluorescent micro/nano-elements. Exquisite true 3D geometry and high-quality morphology (average roughness as low as ~5 nm) were realized for protein-based 3D WGM microlasers by improved FsLDW (higher viscosity of aqueous protein ink, “conformal” scanning mode, optimized laser processing parameters) and efficient “self-smoothing” effect. Consequently, with 532-nm ps-laser pumping light, WGM optical resonation and further lasing actions were successfully achieved in these protein-based 3D-microdisk WGM-lasers in air and even in aqueous environments. The protein-based 3D WGM microlasers without annealing post-processing funtioned satisfactorily and stably in air with ***Q*** round ~2200–2400. Significantly, even in aqueous solutions, the lasing actions still ran well with ***Q*** up to ~3300 and FWHM as low as ~0.18 nm, which was difficlut to realize for previouly reported artificial polymer based WGM microlasers and of great value for their aqueous biorelated applications like biosensing. Other lasing performances including FWHM, FSR, ***I***_th_ were also comprehensively studied in air and in aqueous phase, showing stable lasing actions repeated under proper pumping-light intensities. Optical properties (e.g. RI, absorption spectra) of the protein biopolymers were estimated, which greatly helped to practically introduce this novel protein-based optical material into the library of photonic materials as a new natural-biomacromolecule-based option. Futhermore, the “smart” environment-stimulus-responsive adjusments of central wavelength of lasing spectra were demonstrated using a 60-μm-diameter protein-based 3D WGM microlaser in aqueous environments. A nearly linear blue shift of ~2.59 nm was exhibited responsively to Na_2_SO_4_ concentrations changed from 0 to 5 mmol/L (about 0.4 nm *vs* 0.83 mmol/L per step). Importantly, the other performances (***Q***, FWHM, FSR, peak intensities) were fairly stable during the tuning processes. Therefore, the practical protein-based true-3D WGM microlasers here might bring new opportunities for fields like natrual biopolymer based photonics, tunable “smart” WGM-biolasers, optical biodetection and diagnosis.

## Method

### Preparation of BSA/RhB aqueous ink

BSA (Sigma-Aldrich, A7638) and RhB (CAS No. 81-88-9; Sigma-Aldrich) were dissolved in pure water to obtain FsLDW ink. And as-prepared aqueous ink contained 800–1000 mg/mL BSA and 5 mg/mL RhB, and needed to be quiescent for over 24 hours under 4 °C for sufficient dissolution and combination of BSA and RhB. Here, ultrapure water (18.2 MΩ cm, 25 °C) used in the experiment was from a water purification system from MILLIPORE.

### FsLDW with experimental details

Coverslips were used as substrates of FsLDW-fabrication of micro/nano-devices. Before FsLDW, the coverslips were rinsed sufficiently in acetone, alcohol and phosphate buffer for 15 minutes respectively, and then dried under 95 °C for 10 minutes.

Home-made FsLDW system was built up to fabricate protein-based micro/nano-devices on coverslips. By a high-numerical-aperture (NA = 1.35) oil-immersion objective (60×), a femtosecond laser beam (80-MHz repetition rate, 120-fs pulse width, 800-nm central wavelength; titanium:sapphire laser of Spectra Physics 3960-X1BB) was tightly focused in the ink. A piezo stage with 1-nm precision (PI P-622 ZCD) was used to vertically move the sample, simultaneously, the two-galvano-mirror set was integrated in the FsLDW system for laser beam’s horizontal scanning. Additionally, the fluctuation of the laser pulse energy had to be lower than 0.5 % to achieve a high homogeneity of the voxel sizes that greatly influence the self-smoothing effect, and resluted ultrahigh surface smoothness^3^,^4^,^5^. So, the local temperature surrounding the laser should be maintained at 23+/−0.2 °C and an output feedback system was used.

For “conformal”-scanning 3D WGM microdisks, computer processing data to control 3D scanning of FsLDW were obtained by Visual Basic for more convinient design of scanning mode. And 3D shapes of other micro/nano-structures except 3D WGM microdisks were designed by 3Ds Max and converted to FsLDW-processing data here. Additionally, since it was easy to be dry by evaporation, the high-viscosity BSA/RhB aqueous ink needed to be well sealed in a small polydimethylsiloxane (PDMS) based champer during FsLDW fabrication. In the end, protein-based micro/nano-devices were obtained on the coverslip substrate after laser processing and water-rinsing.

### Fluorescent optical microscopy characterization

The FsLDW-fabricated BSA/RhB micro/nano-structures were observed in air and in water as-needed with a Motic BA400 upright microscope, equipped with 10 × /0.25 and long-focal-distance 40 × /0.60 objective lenses. Here, for clear exhibition of fluorescent images of the 532-nm-excited BSA/RhB micro/nano-hydrogels, sub-565-nm filtering slice was utilized to filter 532-nm pumping laser light out.

### Confocal microscopy characterization

The confocal microscope (Olympus FluoView FV1000) was used here for detailed characterization of BSA/RhB micro/nano-hydrogels by layer-by-layer scanning to obtain 2D and 3D confocal microscopic images. BSA/RhB micro/nano-structures were characterized in air and in water as-needed. 532-nm light was chosen to excite the fluorescence of BSA/RhB gain medium. Grey-scale maps were obtained by confocal microscopy for clear observation of fluorescent RhB's 3D-distribution in Fig. 2a,b. And red in confocal microscopic fluorescent images (Fig. 2c,d) was false color by computer to simulate the fluorescence from RhB.

### SEM characterization

A LEO 1550 FEG SEM was applied to character protein micro/nano-devices at acceleration voltages of 5 kV. Such high BSA concentrations and fully optimized FsLDW processing parameters resulted in high-degree crosslinking and enough solidity of protein micro/nano-devices. So, samples were allowed to air dry directly after pure-water rinsing for faithful SEM characterization of 3D geometry and surface morphology without chemical post crosslinking treatment. Air dry needed to be longer than 4 hours, after which the samples were sputter-coated with ~10-nm thick Au film for SEM characterization.

### AFM characterization

The AFM characterization (Veeco NanoScope) was operated in air to figure out the morphology of series of FsLDW-fabricated BSA/RhB micro/nano-hydrogels dried in air. Tapping mode was adopted to avoid damage to BSA/RhB micro/nano-hydrogels and impact on results by the AFM tip. For Fig. 3e, upper parts of 3D WGM microdisks, that is, 2D microdisks, were FsLDW-fabricated without cylindrical basements for convenient AFM scanning. Importantly, the upper part of a 3D WGM microdisk here was divided into two parts with different scanning mode as described in Fig. 3d. In Fig. 3e, horizontally layered scanning was applied for the inside circles (Ra: 1, 7.2 nm for a 3 × 3-μm^2^ square; 2, 5.3 nm for a 5 × 5-μm^2^ square); the outside rings were scanned by ring-layers parallel and conformal to first-fabricated inside circles' cylindrical side surface (Ra: 1, 12.3 nm for a 5 × 5-μm^2^ square; 2, 13.3 nm for a 5 × 5-μm^2^ square). So the AFM characterization of the upper-part 2D microdisk simultaneously exhibited the different surface quality control by the two scanning modes that the surfaces parallel to scanning layers had much higher quality.

### UV-vis spectra characterization

Relatively dilute solutions of 400-mg/mL BSA with 2-mg/mL RhB and 400-mg/mL BSA with 20-μL/mL photosensitizer 1173 (Ciba DAROCUR 1173, a colorless and transparent water-soluble UV photosensitizer, 2-Hydroxy-2-methyl-1-phenyl-propan-1-one, CAS No. 7473-98-5) were respectively spin-coated on galss slices with 1500 rpm for 30 seconds and followed 6-minute UV exposure to obtain films with similar thickness for comparison. Then, the ultraviolet and visible (UV-vis) spectrophotometer (SHIMADZU UV-2550) was used to measure the UV-vis absorption spectra of BSA hydogels in air to demonstrate optical transparence.

### PL spectra tests

After being frequency-doubled, a picosecond 532-nm laser beam was obtained from a 1064-nm laser (Guoke Amberpico-Q-2000; pulsewidth, 15 ps; repetition rate, 50 KHz) for optical pumping. An attenuator and a shutter were used to control pumping light intensity and pumping interval respectively. By a 16-mm-focal-distance convex lens, the pumping laser beam was focused onto samples. To obtain the PL spectra, a fiber optic spectrometer was used to collect the PL light scattered from side surface of protein-based 3D WGM microlasers. Meanwhile, a CCD (charge coupled devices) was integrated in the self-made WGM-lasing testing system for lively observation of microdevices that were being tested. The pumping laser spot was estimated of ~170-μm diameter and ~22687-μm^2^ area for estimation of pumping power intensity (Figure S3).

The corresponding pumping intensities in Fig. 4a,d and Fig. 5a were as followed, Fig. 4a: 1, 0.194; 2, 0.216; 3, 0.229; 4, 0.401; 5, 0.551; 6, 0.670; 7, 0.771; 8, 0.877; 9, 1.031. (Unit, μW/μm^2^) Fig. 4d: 1, 0.577; 2, 1.252; 3, 1.477; 4, 1.825; 5, 2.147; 6, 2.614; 7, 3.134; 8, 3.570; 9, 3.976. (Unit, μW/μm^2^) Fig. 5a: 1, 1.168; 2, 1.490; 3, 1.609; 4, 2.071; 5, 2.676; 6, 3.262; 7, 3.844. (Unit, μW/μm^2^)

## Additional Information

**How to cite this article**: Sun, Y.-L. *et al.* Protein-Based Three-Dimensional Whispering-Gallery-Mode Micro-Lasers with Stimulus-Responsiveness. *Sci. Rep.* **5**, 12852; doi: 10.1038/srep12852 (2015).

## Supplementary Material

Supplementary Information

Supplementary Information

Supplementary Information
