# Supplementary material for: Protein-Based Three-Dimensional Whispering-Gallery-Mode Micro-Lasers with Stimulus-Responsiveness
Source: Sci Rep. 2015 Aug 4;5:12852. doi: 10.1038/srep12852 (PMC4523880; doi:10.1038/srep12852)
Supplement: Supplementary Information [file srep12852-s1.pdf]

Supplementary information

Protein-Based Three-Dimensional Whispering-Gallery-Mode Micro-Lasers with Stimulus-Responsiveness

Yun-Lu Sun[1], Zhi-Shan Hou[2], Si-Ming Sun[1], Bo-Yuan Zheng[1], Jin-Feng Ku[1], Wen-Fei Dong[1], Qi-Dai Chen[1] & Hong-Bo Sun[1,2]

[1]State Key Laboratory on Integrated Optoelectronics, College of Electronic Science and Engineering, Jilin University, 2699 Qianjin Street, Changchun 130012,China.

[2]College of Physics, Jilin University, 119 Jiefang Road, Changchun, 130023, China.

Correspondence and requests for materials should be addressed to Prof. Hong-Bo Sun (hbsun@jlu.edu.cn)

Keywords: (Biomacromolecules, protein hydrogel, whispering-gallery-mode micro-laser, stimuli-responsiveness, femtosecond laser direct writing)

Video 1. The animation of the FsLDW fabrication of a 3D WGM microdisk with simply layered scanning mode.

Video 2. The animation of the FsLDW fabrication of a 3D WGM microdisk with conformal scanning mode.

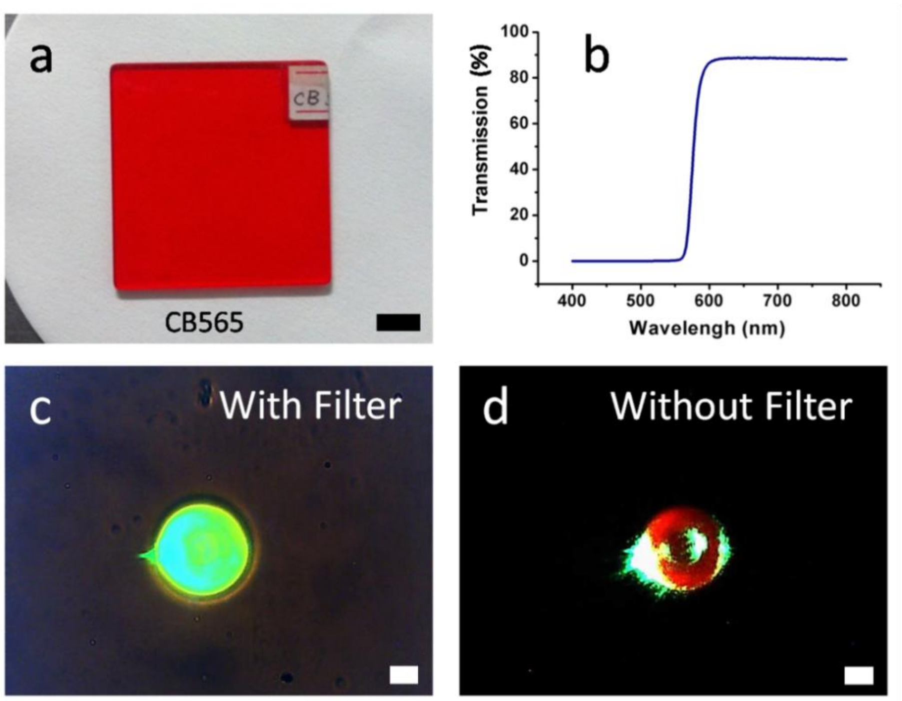

Figure S1. (a) The sub-565-nm filter slice used in fluorescent optical microscopy. Scale bar, 1 cm. (b) The transmission spetrum of the sub-565-nm filter slice. (c) A fluorescent optical microscopy image after filtering with the sub-565-nm filter slice in (a). Scale bar, 10 μm. (d) An original fluorescent optical microscopy image without filtering. Scale bar, 10 μm.

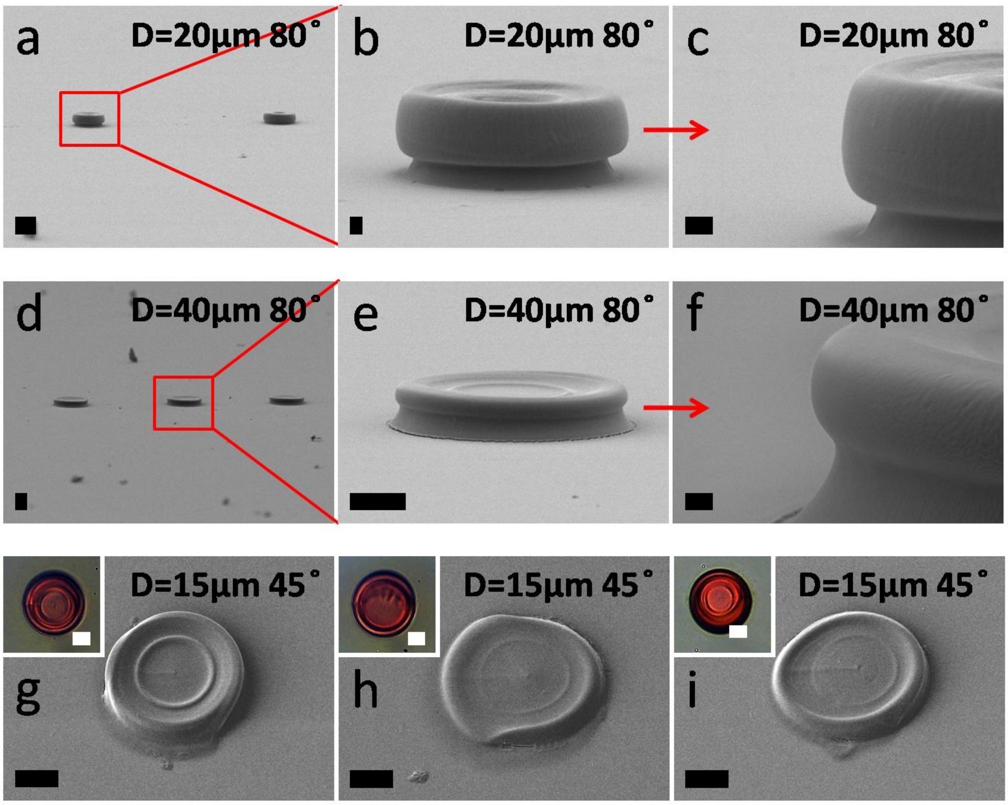

Figure S2. (a-c) Side-view SEM images of a 80 ° tilted 20-μm-diameter (4-μm designed thickness) protein-based 3D WGM microdisk FsLDW-fabricated with higher laser power (~ 32 mW), resulting into a little bloated 3D geometry.Scale bar, (a) 10 μm, (b) 1 μm, (c) 1 μm. (d-f) Side-view SEM images of a 80 ° tilted 40-μm-diameter (4-μm designed thickness) protein-based 3D WGM microdisk FsLDW-fabricated with lower laser power (~ 20 mW), resulting into a little shinked 3D geometry. Scale bar, (d) 10 μm, (e) 10 μm, (f) 1 μm. (g-i) multi-occurrent and high risk of collapse of protein-based 3D WGM microlasers fabricated with simply layered scanning mode and the same other FsLDW factors, especially for the true-3D overhanging parts. Scale bar, 10 μm.

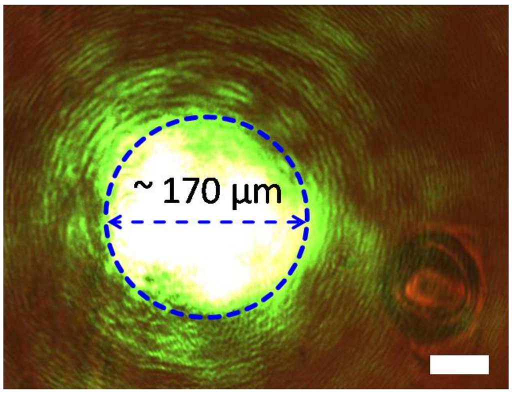

Figure S3. Optical image of 532-nm pumping laser focal spot on a sample slice. Scale bar, 50 μm.

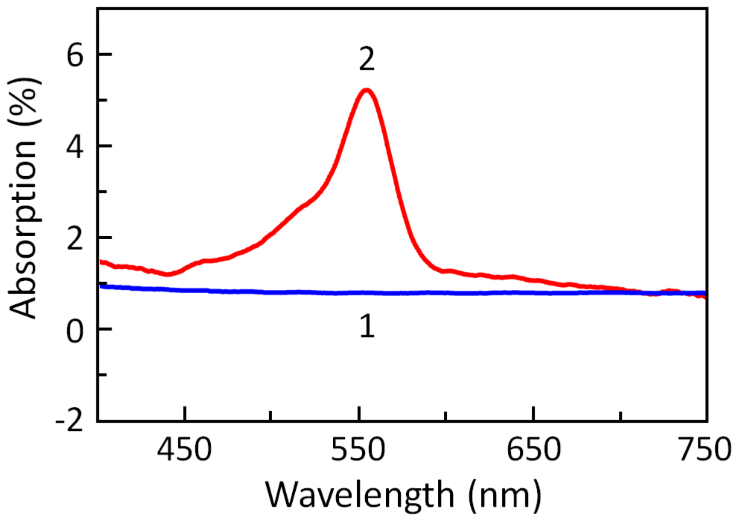

Figure S4. UV-vis absorption *vs* wavelength of BSA/RhB composite hydrogel films with RhB or 1173 as photosensitizers obtained by UV polymerization. 1, UV-vis absorption spectrum of BSA/1173 composite film. 2, UV-vis absorption spectrum of BSA/RhB composite hydrogel film.

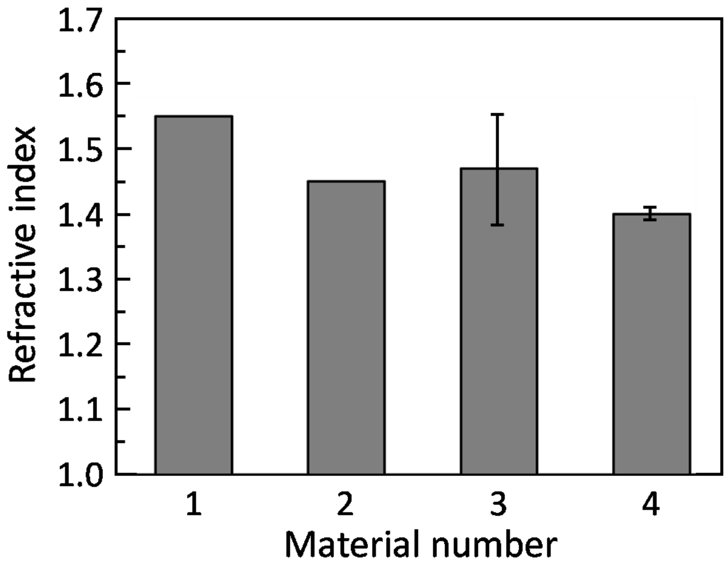

Figure S5. Estimated RI of BSA based micro/nano-hydrogels by FsLDW. 1, RI of BSA/MB hydrogel in air, ~ 1.55.[3, 4] 2, RI of BSA/MB hydrogel in pure water, ~ 1.45.[3, 4] 3, RI of BSA/RhB hydrogel here in air, ~ 1.47. 4, RI of BSA/RhB hydrogel here in pure water, ~ 1.40.

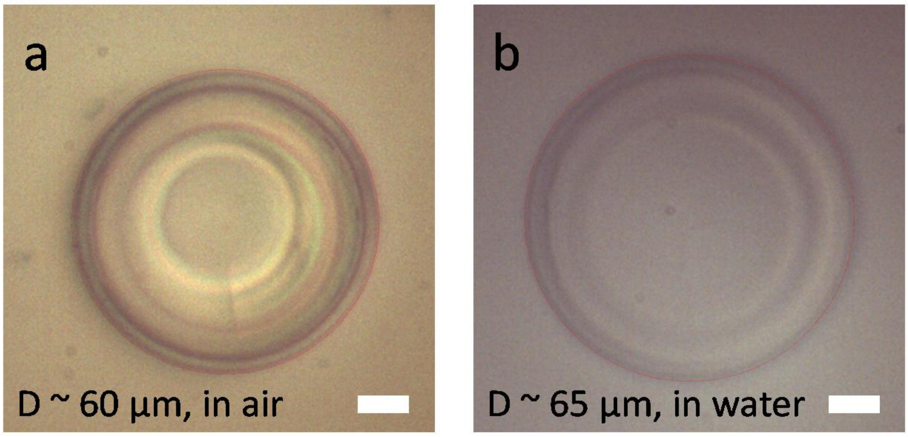

Figure S6. Optical microscopic images in air (a) and in pure water (b) of a BSA/RhB 3D WGM microdisk with designed diameter of 60 µm. Scale bar, 10 µm.

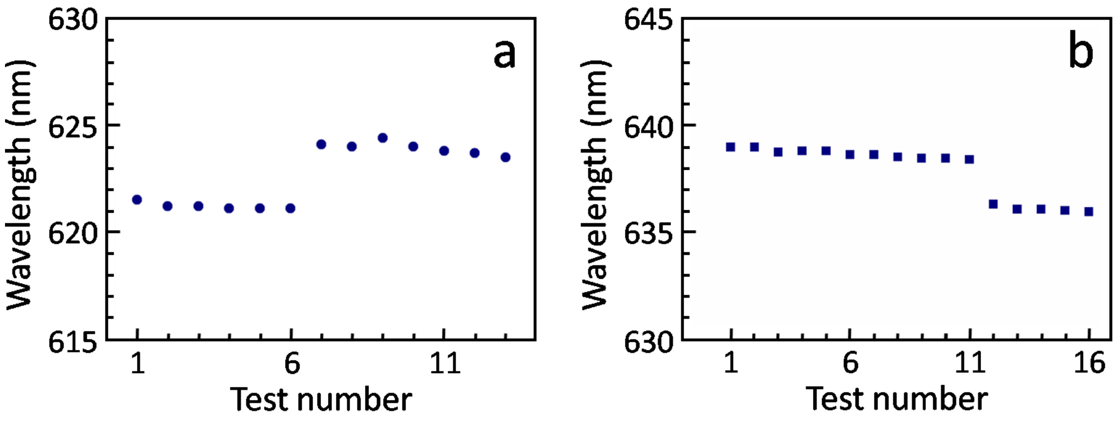

Figure S7. (a) Central wavelengths corresponding to the highest peaks of lasing spectra of different test number for a 30-μm-diameter protein-based 3D WGM microlasers in air in Figure 4 a and c. Pumping intensity of test number 10, 1.34 μW/μm^2, 11, 1.55 μW/μm^2, 12, 1.75 μW/μm^2, 13, 2.10 μW/μm^2. (b) Central wavelengths corresponding to the highest peaks of lasing spectra of different test number for a 40-μm-diameter protein-based 3D WGM microlasers in air in Figure 4 d and f. Pumping intensity of test number 10, 3.13 μW/μm^2, 11, 3.57 μW/μm^2, 12, 3.98 μW/μm^2, 13, 4.71 μW/μm^2, 14, 5.21 μW/μm^2, 15, 6.17 μW/μm^2, 16, 6.27 μW/μm^2.

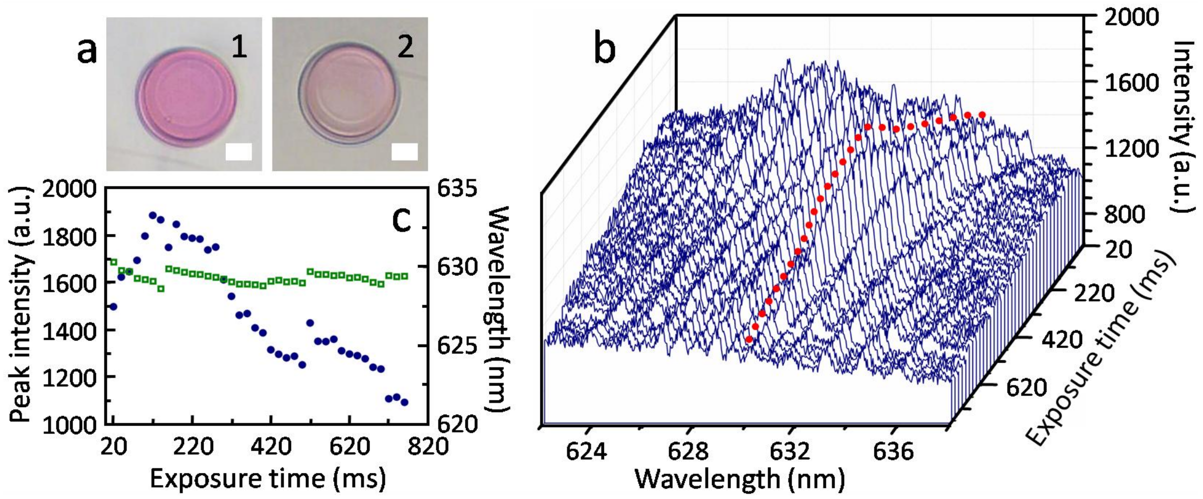

Figure S8. PL performance changed along with increasing exposure time in water. (a) Optical microscopic images of the tested BSA/RhB-based 60-μm-diameter WGM 3D microdisk (1) before and (2) after multiple 532-nm exposures in water (1.5-μW/μm^2 pumping light intensity). Scale bar, 10 μm. (b) Lasing spectra under multiple exposures in water. (c) Increasing-exposure-time-induced change of intensity (blue dots) and wavelength (green squares) of the lasing peak at ~ 630 nm marked in (b).

In Figure S8, 532-nm light with a relatively low intensity of about 1.5 μW/μm^2 was used to repeatedly pump a BSA/RhB-based 60-μm-diameter WGM 3D microlaser in water. After about 800-ms exposure in total, RhB loaded in protein hydrogel obviously faded via photobleaching, while the device structure and morphology were not changed (see Figure S8 (a)). Accordingly, the PL lasing spectra were weakened gradually along with multiple exposures (20ms per time) as shown in Figure S8 (b). For one certain lasing peak at ~ 630 nm, the intensity decreased and wavelength were essentially unchanged during the multiple exposures (Figure S8 (c)). It proved that the protein matrix material was tolerant to relatively long-time exposure with proper pumping intensity, and photobleaching of RhB might be the main reason impacting the lasing performance during long-time or multiple exposures.

More importantly, in series of experiments in Figure S7, S8 and Figure 4 in the manuscript, the position of a certain lasing peak was approximately constant during multiple exposures even if PL lasing performance was weakened via RhB photobleaching. So, RhB photobleaching might have less influence on the ion-strength-responsive peak shifts of lasing spectra in Figure 5 (d)-(f) in the manuscript.

Spectrum number	Na_2SO_4 concentration (mol/L)	Peak wavelength (nm)	FWHM (nm)	***Q*** (Wavelength/FWHM)	FSR (nm)	Peak intensity (a.u.)
1	0	611.14	0.25	2444.6	1.31	1443
2	0	611.07	0.29	2107.1	1.33	1441
3	0	611.03	0.24	2546.0	1.27	1414
4	8.33×10^{-4}	610.77	0.28	2181.3	1.28	1526
5	16.67×10^{-4}	610.12	0.41	1488.1	1.31	1390
6	25.00×10^{-4}	609.61	0.181	3368.0	1.31	1393
7	33.33×10^{-4}	609.42	0.256	2380.5	1.32	1340
8	41.67×10^{-4}	608.87	0.256	2382.1	1.32	1349
9	50.00×10^{-4}	608.55	0.183	3325.4	1.31	1327

Table S1. Specific data of several main performances of the protein-based 3D WGM microlaser in Figure 5 d, e, and f corresponding to test number during tuning processes.

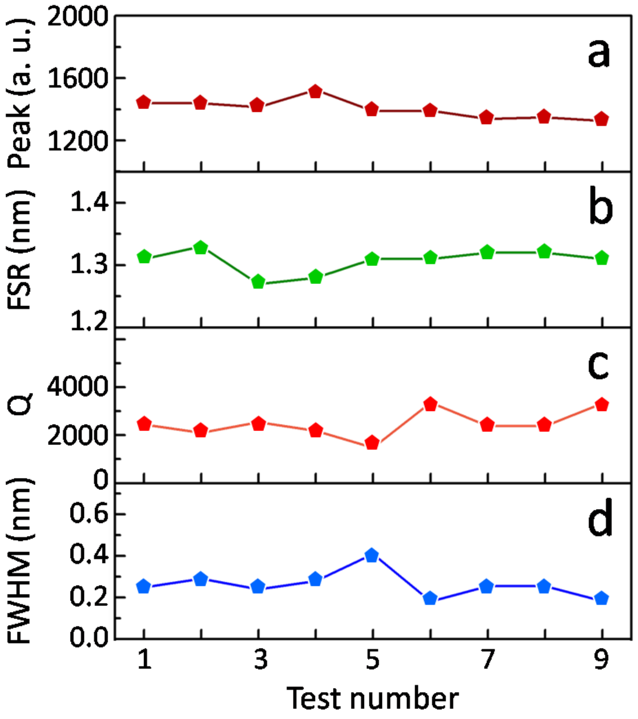

Figure S9. Several main performances of the protein-based 3D WGM microlaser in Figure 5 d, e, and f corresponding to test number during tuning processes in Table S1. (a) The highest peaks of lasing spectra *vs* different test numbers. (b) FSR of lasing spectra *vs* different test numbers. (c) ***Q*** of lasing spectra *vs* different test numbers. (d) FWHM of lasing spectra *vs* different test numbers.
